# The royal society of medicine open section’s medical humanities conference: physician resilience and compassionate practice

**DOI:** 10.1080/17571472.2015.1135652

**Published:** 2016-02-24

**Authors:** Amelia Martin

**Affiliations:** ^a^ University of Bristol, Bristol, United Kingdom

The RSM Open Section’s medical humanities conference held on the 5th of October set out to explore the relationships between literature, the humanities and healthcare. Clinician resilience and compassionate care were explicit themes for the day. Professor Deborah Bowman, the voice of Radio 4’s *Inside Ethics Committee* launched the conference by exploring the parallels between Medicine and the theatre. She examined the work of Samuel Beckett, helping the audience to consider the ‘altered state’. Beckett’s line ‘to be what I always am, so changed from what I was’ [1], highlights ‘Consistency for person’ and how we, as healthcare professionals, ask about change. Bowman drew lessons from the theatre to be applied in practice to ‘attend to the objective and subjective, to attend to the abstract not in the room and to attend to the self and other’. These wider considerations can help healthcare professionals to hold onto compassion in medical practice which sometimes can be lost through the pressures of working within systems. Professor Bowman concluded that in order to ask someone to practise in a compassionate way, like an actor in a play, clinicians need rehearsal in order to be able to give voice to values.

Following on with a theme of theatre and human connection were Professor Steven Bergman, author of the internationally renowned ‘The House of God’ [2] written under the pseudonym of Samuel Shem (Shem), and his co-writer and wife Dr Janet Surrey, a clinical psychologist. They discussed their successful off-Broadway play Bill W and Dr Bob; a play that explored the healing dialogue between the men who founded Alcoholics Anonymous. They then moved to reflect on 15 years of co-work to bring men and women together to explore relational qualities. Discussing their book ‘We have to Talk: Healing Dialogues between women and men’ [3] they highlighted common gender related miscommunications that can occur and how they may be avoided. The lessons found in their latest book ‘The Buddha’s Wife’ [4] include how to reconsider, ‘How will *I* solve this’ to ‘How can *we* get out of this’ (verbatim quote) and through developing relationships with people, one moves from *single* to *we*, which in turn allows greater care to evolve.

Former GP and international essayist Iain Bamforth challenged the assembled audience with an exploration of two early twentieth-century French doctors who wrote novels, namely Dobler and Celine. They were doctors in society and illustrate the interests, prejudices and even the sense of disgust that we might discover in ourselves.

Shem built on many earlier themes in his talk on physician resilience and compassionate practice. The resounding message from Shem for healthcare professionals: ‘the risk of isolation and the healing power of good connection’. Rather than *communicate* with patients (we can communicate with machines), we should attempt to *connect*. Compassionate practice arises from good connections, community and vision. In Shem’s new book, he moves from exploring how to stay human in hospital Medicine in ‘The House of God’ [2] to how to be a doctor in the community in ‘The Spirit of the Place’. [5] His character sets out to heal the town, and in the process of this the town heals him.

The messages from the conference were strong and lasting. In order to connect, we must show compassion and give voice to our values. The speakers highlighted that the intersection between the arts and medicine has much to offer in helping us to do so – using the humanities as a vehicle for reflection on values and recognising that much of the content of the humanities has intrinsic value – even as historical data. A key learning point of relevance in all areas of practice, is to recognise when systems lose sight of the person at the centre of care. The solution is to strive towards continual improvement to deliver safe effective healthcare with human values at the heart of medical practice.

## Notes on contributor

***Amelia Martin*** is a student representative for The Open Section of the RSM. The Open Section is the section concerned with the interface between clinical and public interests. For more information visit www.rsm.ac.uk.
